# Validating a Japanese Version of the Athlete Psychological Strain Questionnaire

**DOI:** 10.1186/s40798-021-00385-9

**Published:** 2021-12-11

**Authors:** Yasutaka Ojio, Asami Matsunaga, Shin Kawamura, Masanori Horiguchi, Goro Yoshitani, Kensuke Hatakeyama, Rei Amemiya, Ayako Kanie, Rosemary Purcell, Simon M. Rice, Chiyo Fujii

**Affiliations:** 1 Department of Community Mental Health and Law, National Institute of Mental Health, National Center of Neurology and Psychiatry, 4-1-1 Ogawa-Higashi, Kodaira, Tokyo, 187-8553 Japan; 2 Japan Rugby Players’ Association, Tokyo, Japan; 3 Faculty of Health and Sports Sciences, University of Tsukuba, Tsukuba, Ibaraki Japan; 4 National Center for Cognitive Behavioral Therapy and Research, National Center of Neurology and Psychiatry, Tokyo, Japan; 5 Orygen, Parkville, VIC Australia; 6 Centre for Youth Mental Health, The University of Melbourne, Melbourne, VIC Australia

**Keywords:** Mental health, Screening, Rugby player, APSQ, Help-seeking

## Abstract

**Background:**

There is increasing international interest in clinical practice and research related to mental health in the international sports society. The athlete-specific psychological distress assessment tool that addresses potential mental health needs can help promote early detection and recovery of mental illness, as well as physical illnesses. Currently, little is known about the applicability of the useful assessment tool for Japanese elite athletes. The Athlete Psychological Strain Questionnaire (APSQ) is a brief, effective and reliable screening tool to identify early signs of athlete-specific distress and potential mental health symptoms. We examined the applicability and reliability of a Japanese version of the APSQ (APSQ-J) in a Japanese elite athlete context. Further, we examined the construct validity of the APSQ-J.

**Methods:**

We collected web-based anonymous self-report data from 219 currently competing Japanese professional male rugby players. A two-stage process was conducted to validate the factor structure of the APSQ-J using exploratory factor analysis (EFA) in a randomly partitioned calibration sample and confirmatory factor analysis (CFA) in a separate validation sample. Cronbach’s alpha is used to assess internal consistency. Pearson product-moment correlation coefficients were calculated to determine if the APSQ-J was significantly associated with measures of psychological distress and well-being using Kessler-6 (K6) and the WHO-5 Well-Being Index, respectively.

**Results:**

We identified a one-factor structure for the APSQ-J. Confirmatory factor analysis supports this one-factor model, revealing good model fit indices. The standardized path coefficients for each of the items were *β* = 0.41–0.83 (*p* < 0.001). A Cronbach’s alpha of 0.84 was obtained for the APSQ-J. The APSQ-J demonstrated significant correlations with the K-6 (*r* = 0.80, *p* < 0.001) and WHO-5 (*r* = −0.58, *p* < 0.001).

**Conclusion:**

The APSQ-J can be an appropriate and psychometrically robust measure for identifying athlete-specific distress in elite athletes in Japan. Widely disseminating and utilizing this scale in Japanese sports society may support athletes' mental health via early detection of symptoms of psychological distress.

**Supplementary Information:**

The online version contains supplementary material available at 10.1186/s40798-021-00385-9.

## Key points

- Athlete Psychological Strain Questionnaire (APSQ) is an athlete-specific psychological distress assessment tool that addresses potential mental health needs.
- Current findings from the sample of Japan Rugby Top League players supported the factor structure, validity, and reliability of the Japanese version of the APSQ (APSQ-J) scale, with a one-factor structure, indicating the higher score, the worse the psychological distress and strain.
- APSQ-J is a brief and effective evaluation tool that facilitates the development and social implementation of mental health support for elite athletes in Japan.

## Background

Recent research and clinical practice for mental health in elite athletes has increased, similar to the trajectory for physical health and injury care. Most of the findings have come from the USA [1, 2], European countries [3–6], and Australasia [7, 8], with studies from Asia still limited [9, 10]. These findings consistently demonstrate that mental health symptoms are common in elite athletes [11]. Contributing factors include athlete-specific factors (e.g. physical injury, poor performance, competition for selection, career transition) and general factors, such as stressful life events and inadequate social support [6, 8, 9, 11, 12]. Several statements presented by international sports organizations and experts call for mental health care systems and environmental improvements for athletes [11, 13–20]. Mental health status affects competitiveness, as well as non-competitive daily living, and delays in support and care for poor mental health can worsen symptoms [21]. An athlete-specific mental health assessment tool can help facilitate early detection and recovery from mental ill-health, as with physical illness.

The Athlete Psychological Strain Questionnaire (APSQ) is a mental health assessment tool that addresses these potential needs specifically within an elite sports context [22, 23]. The APSQ is a 10-item scale developed by Rice and colleagues using survey data on Australian male athletes [22] and also validated with female elite athletes [23]. The authors created the item pool from mental health research findings related to athletes, and the preliminary scale, validated in male athletes, consisted of 10 items loading onto three factors, namely Self-Regulation, Performance, and External Coping.

The APSQ is included as the triage measure in the Sports Mental Health Assessment Tool-1 (SMHAT-1), a mental health screening procedure developed by the International Olympic Committee (IOC) to better support the mental health of athletes and stimulate more international research [24]. At present, little is known about the applicability of the APSQ to Japanese elite athletes. The development of a Japanese version (the APSQ-J) may promote mental health research and practice for elite athletes in Asia, including Japan. Therefore, this study aims to develop and validate a Japanese version of the APSQ in a sample of Japanese male professional rugby players. In addition, the other aim is to compare the means of APSQ-J with the original APSQ from the sample of Australian male elite athletes to understand how similar they are.

## Methods

### Study Design and Setting

The current study employed a web-based cross-sectional design. We followed the Strengthening the Reporting of Observational Studies in Epidemiology (STROBE) guidelines for observational cross-sectional studies (Additional file 1) [25]. We distributed the survey weblink (URL) to all players in the Japan Rugby Players’ Association through one or two team representatives participating in regular meetings. The potential participants were invited to complete the anonymous survey. They were informed about the survey process, including the purpose of the study, data collection procedures, and the consequences of participating or not participating via the cover page of the questionnaire. Consenting participants went on to complete the questionnaire, which took less than 10 min. The participants were provided with individual one-time access to the survey using IP address filtering access to a tablet or laptop computer to complete the survey. The cross-sectional data were collected shortly before the start of the off-season from December 2020 to February 2021.

### Participants

We collected data from a total of 612 rugby players (565 Japanese players and 47 foreign players) registered with the Japan Rugby Players Association. The participants were all aged 18 years and over and belonged to the Japan Rugby Top League. The current survey was available in both Japanese and English. No exclusion criteria were applied. Overall, 227 of the 612 players agreed to complete the survey (response rate: 37.1%). The response rate of this survey was consistent with other mental health surveys in Japan [26]. We analysed 219 out of 227 participants born in Japan as the target population in the current study. All investigators received the learning course on research ethics, and this study was approved and facilitated by the Research Ethics Committee at the National Center of Neurology and Psychiatry (approval number: A2020-058).

### Measurements

#### The Athlete Psychological Strain Questionnaire (APSQ)

The APSQ is a brief, self-report questionnaire specific to the elite sporting context, comprised of 10 items (e.g. ‘During the past 4 weeks, I could not stop worrying about injury or my performance’) scored on a 5-point scale (from ‘none of the time’ (1) to ‘all of the time’ (5)). A total score ranging from 10 to 50, with higher scores representing more psychological distress, is calculated by summing the answers on the 10 items [22]. A more recent study has shown that scores of ≥ 15, ≥ 17 and ≥ 20 represent moderate, high and very high levels of athlete-specific distress [23]. In the preliminary stage of developing the original version of APSQ [22], a two-step approach was used in a sample of 1007 currently competitive Australian elite male athletes from professional sports (*M* = 23.67, SD = 4.16). The exploratory and confirmatory factor analysis and tests of differential item functioning were conducted with the samples randomly partitioned into calibration (*n* = 497) and validation (*n* = 510) samples. Exploratory factor analysis, with parallel analysis, conducted on the calibration sample supported a second-order with three-factor model. The subscales included Self-Regulation, Performance, and External Coping domains, accounting for 50.44% of total scale variance. In the second-order model, the path coefficient from the upper factor “Athlete psychological strain” to each factor was 0.8 or more. In the confirmatory factor analysis, excellent model goodness-of-fit indicators were provided. The mean score for the 10 items among the Australian male athletes was 14.67 (SD = 5.47).

For this study, the original APSQ was translated into Japanese by the first author (Y.O.). The professional athletes (S.K. and K.H.) in this research team modified the terms and sentences to improve the readability for athletes. A bilingual English speaker then produced a back-translation. Finally, the back-translated version of the APSQ was confirmed and approved by the researchers who had originally developed the APSQ (S.M.R and R.P).

#### Other Measurements

We used the Kessler-6 (K6) and the WHO-5 Well-Being Index, widely used measures of psychological distress and well-being to assess convergent validity with the APSQ-J. Previous studies have shown that the performance of the K6 in screening mood and anxiety disorders, as assessed by the areas under the receiver operating characteristic curves (AUCs), was excellent [27], with values as high as 0.86, 0.89, and 0.94 from US [28], Australian [29], and Japanese general samples [30], respectively. In the K-6, the scores are categorized to indicate the respondents’ mental health status over the previous 30 days. Responses to items are made on a 5-point scale. The K-6 was developed and validated based on many epidemiological surveys and is widely used as a screening tool in assessing treatment progress in common mental disorders such as anxiety and depression in people in the general community [31]. Cronbach’s α was 0.91 in the present sample for the K6. The WHO-5 Well-Being Index is a 5-item self-report scale that assesses positive aspects of mental health (i.e. “I have felt cheerful and in good spirits”) over the previous 2 weeks [32, 33]. The raw score is calculated by totalling the figures of a 5-point scale (i.e. 0 = “at no time”; 5 = “All of the time”). The score ranges from 0 to 25, with 0 representing the worst possible and 25 representing the best possible well-being. A score below 13 indicates poor well-being and is an indication for assessing for depression according to the ICD-10. Cronbach’s α was 0.90 in the present sample for the WHO-5.

#### Background Information/Demographics

The background information and demographic survey items included age, country of birth, educational attainments, marital status, the number (if any) of dependent children, residential status, the national team's experience, and playing status for the last season.

### Statistical Methods

Descriptive statistics were used to characterize the sample. We conducted a two-stage process to validate the factor structure of the APSQ-J using exploratory factor analysis (EFA) in a randomly partitioned calibration sample and confirmatory factor analysis (CFA) in a separate validation sample. EFA was undertaken to determine the underlying factor structure of the APSQ-J. To determine which items belonged to each factor, we extracted items if they loaded ≥ 0.3. In addition, we examined the number of factors based on scree plots. We adopted the oblique rotation (promax) with principal factor method. In CFA, we evaluated the fit of the model with the data using the chi-squared statistic (CMIN), root mean square error of approximation (RMSEA), and comparative fit index (CFI). According to conventional criteria, an excellent fit would be indicated by CMIN/df < 2, RMSEA < 0.05, CFI > 0.97, while CMIN/df < 3, RMSEA < 0.08, CFI > 0.95 demonstrates an acceptable fit [34]. After confirming the factor structure, we examined the internal consistency and convergent and divergent validity. To evaluate internal consistency, the Item-test, Item-rest correlation and Cronbach’s alpha was calculated. In terms of convergent validity, Pearson product-moment correlation coefficients were calculated to determine if the APSQ-J correlated significantly with the K-6 and WHO-5. Therefore, we determined convergent and divergent validity through examining patterns of statistically significant correlations with the K-6 (convergent validity evidenced by significant positive associations) and the WHO-5 (divergent validity evidenced by significant negative associations). We conducted a one-sample t test for comparing the current data to the means reported in developing the APSQ paper from the 1007 currently competing Australian elite male athletes [22]. For the effect size of the mean difference between the two countries in each assessment point, through computing Cohen’s d, which was graded as 0.20 = small, 0.50 = medium, 0.80 = large. All analyses were conducted with Stata version 16 (StataCorp LLC, College Station, TX, US).

## Results

We analysed 219 players born in Japan. The demographic characteristics of the participants are shown in Table 1. Almost half were 25–29 years old; the overwhelming majority had graduate university educational attainment and one in five players reported experience in the national team.

**Table 1:** Demographic characteristics of the study participants

	% (*n*)
*Age at survey*	
20–24	27.85 (61)
25–29	45.66 (100)
30–34	21.46 (47)
35–	5.02 (11)
*Educational attainment*	
High school	1.82 (4)
Four-year college or university	97.3 (213)
Postgraduate college (or higher)	0.91 (2)
*Marital status*	
Married	47.49(104)
Never married	51.60 (113)
Divorced or widowed	0.91 (2)
*Child living in household*	
No	75.34 (165)
Yes	24.66 (54)
*Residential status*	
Living alone	21.92 (48)
Living with family and/or partner	47.95 (105)
Dormitory	30.14 (66)
*Experience of national team*	
No	80.37 (176)
Yes	19.63 (43)
*Playing status of last season*	
As an active member	34.25 (75)
As a reserve member	28.77 (63)
No play	36.99 (81)

**Fig. 1:**
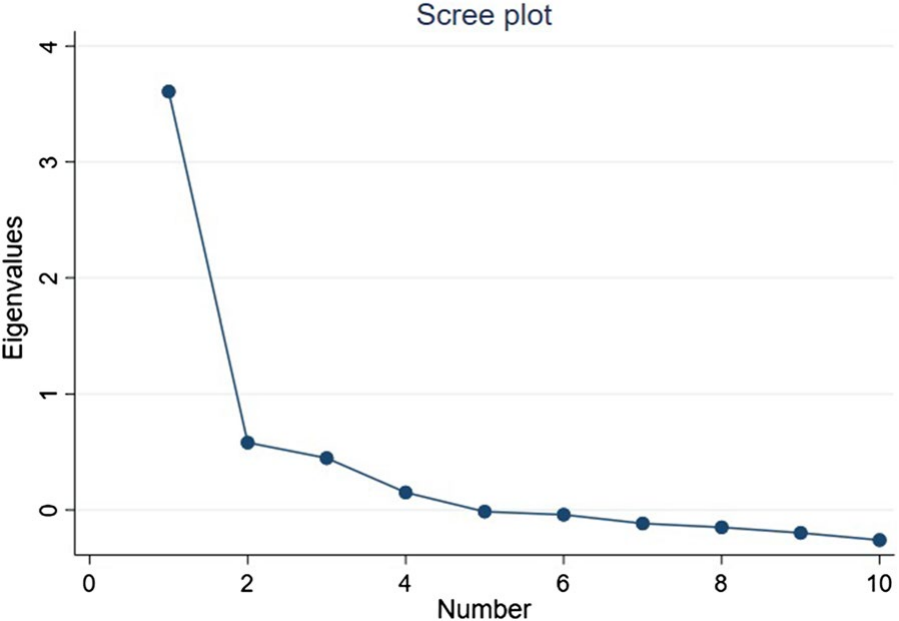
Scree plots and parallel analysis for APSQ-J

In EFA, considering the results of the scree plots (Fig. 1), we employed a one-factor structure as optimal. Factor loadings of the items are shown in Table 2. When the one-factor structure was employed, the factor loading values for all the items were ≥ 0.3.

**Table 2:** Factor loadings for APSQ-J items

Item no	Statement	Factor loadings
1	It was difficult to be around teammates	0.60
2	I found it difficult to do what I needed to do	0.75
3	I was less motivated	0.76
4	I was irritable, angry or aggressive	0.58
5	I could not stop worrying about injury or my performance	0.63
6	I found training more stressful	0.74
7	I found it hard to cope with selection pressures	0.61
8	I worried about life after sport	0.49
9	I needed alcohol or other substances to relax	0.31
10	It was difficult to be around teammates	0.37

**Fig. 2:**
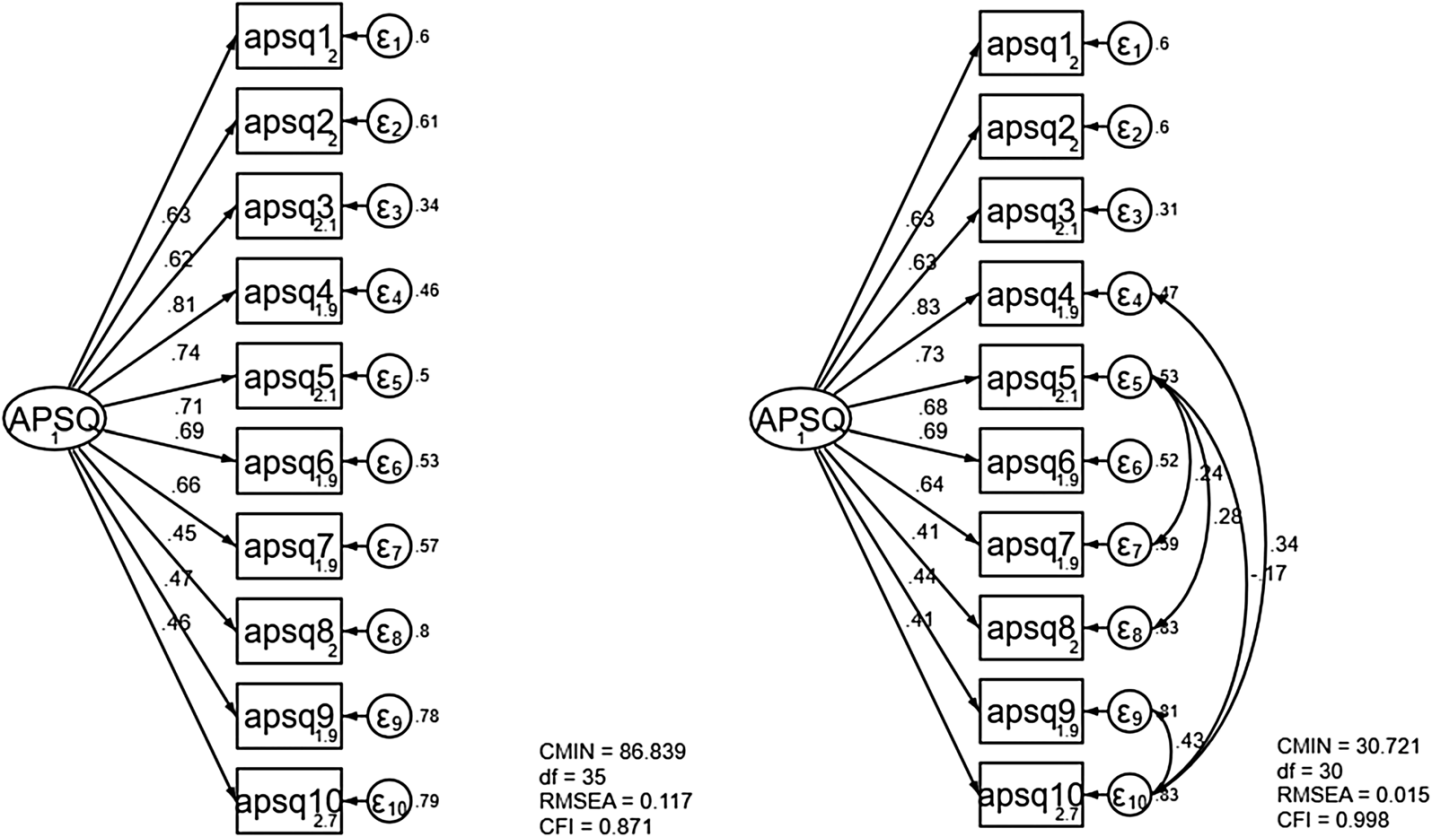
Standardized regression weights for the APSQ-J one-factor structure. Abbreviation: Chi-square statistics (CMIN); root mean square error of approximation (RMSEA); comparative fit index (CFI); Tacker-Lewis fit index (TLI)

The results of CFA are presented in Fig. 2. The one-factor model of APSQ-J showed the following model fit indices: CMIN/*df* = 2.48 (*p* < 0.001), RMSEA = 0.117, CFI = 0.871. Along with modification indices which suggest a high parameter change, covariances were added between some of the error variables of items. After the modification, we obtained a final model with excellent fit indices: CMIN/*df* = 1.024 (*p* < 0.001), RMSEA = 0.015; CFI = 0.998. The standardized path coefficients for each item were *β* = 0.41–0.83 (*p* < 0.001).

The Item-test, Item-rest correlation for the 10-items APSQ-J is shown in Table 3. The Cronbach’s alpha (α) of 0.84 was obtained for the APSQ-J. APSQ-J showed a significant strong positive correlation with K-6 (*r* = 0.80, *p* < 0.001) and a significant moderate negative correlation with WHO-5 (*r* = −0.58, *p* < 0.001). The total score for the 10 items was significantly higher than that for Australians (mean = 19.16 (SD = 6.30) vs. mean = 14.67 (SD = 5.47), t = 10.70; df = 1224; *p* < 0.001) in Additional file 1: Table S1. The effect size for the difference was almost large (Cohen's d: 0.76).

**Table 3:** Item-test, item-rest correlation and Cronbach’s alpha of the APSQ-J

Item no	Item-test correlation	Item-rest correlation	*α*
1	0.63	0.54	0.83
2	0.70	0.61	0.82
3	0.78	0.70	0.81
4	0.68	0.59	0.83
5	0.75	0.64	0.82
6	0.74	0.65	0.82
7	0.71	0.59	0.83
8	0.59	0.44	0.84
9	0.45	0.36	0.84
10	0.44	0.39	0.84
Test scale			0.84

## Discussion

This study evaluated the psychometric properties of the Japanese version of APSQ in order to contribute to improving mental health promotion and support for Japanese elite athletes. We confirm the factor structure, validity, and reliability of the scale in Japanese male professional rugby players. The APSQ-J demonstrated a one-factor structure which is different from the three-factor of the original version of APSQ from the factor analysis. The APSQ-J could be considered a useful assessment tool for athlete-specific psychological distress in Japanese elite athletes, with relatively high Cronbach’s alphas, as well as convergent validity with the K-6 and WHO-5.

The Japanese version of APSQ did not match the factor structure of the original version of APSQ. The original version had three factors: Self-regulation, Performance, and External Coping [22, 23], while the Japanese version had one factor. The differences might reflect behavioural or reaction patterns related to psychological distress and mental health that are closely associated with national cultures. For example, Japanese culture has a “tightness” social norm and low tolerance of deviant behaviour to enhance order and social coordination to effectively deal with threats to social cohesion [35]. Emphasizing such harmonious social relationships tends to prefer self-improvement rather than externalization as a coping action [36]. In addition, people in Japan also tend to hesitate to seek help due to a strong influence of stigma compared to Western countries [37, 38]. In addition, this study was a preliminary survey involving only male rugby players which may have further influenced the results. In the Japanese sports society, where there is little research on the mental health of elite athletes, the development of this preliminary scale provides an opportunity for more research in the future.

Our findings suggest that the APSQ-J confirmed a good convergent and divergent validity with the K-6 and WHO-5, respectively. The advantage of using the APSQ-J in elite athlete populations is the ability to detect athlete-specific distress, which can then enable mental health support for affected athletes. The APSQ is a 10-item, sufficiently brief tool highly relevant to the competitive sport context, by its inclusion of items related to injury, training, and selection, as well as mental health-related externalization issues such as alcohol and substance abuse. While K6 focuses on internalized symptoms, the APSQ includes several externalized symptoms in addition to such internalized symptoms. This feature was included as elite athletes conforming to traditional masculinity norms may be more likely to experience and manifest externalized, rather than internalized symptoms, as a sign of poor mental health [11, 39]. Focusing on the response to each item may also help provide practical mental health support for athletes.

The current study showed that the items of APSQ-J are summed as factor to generate a total score, suggesting that the higher the score, the worse the psychological distress and strain. We also demonstrated that the total score in the sample of Japanese male rugby players was higher than that of Australian male elite athletes. Considering the difference of the total score in each country, the next step in developing the APSQ-J will be to further explore the validity of APSQ-J cut-off values, including relative to the original version [23]. Furthermore, the APSQ-J can also be utilized in the Japanese version of SMHAT-1 that is under development by the authors [24].

### Strengths and Limitations of this Research

The major strengths of this study were developing a Japanese version of the APSQ and confirming its psychometric properties. Our efforts are expected to advance mental health research among elite athletes in Japan, which has been delayed in comparison with the USA, European countries and Australia. In translating the scale development process, we coordinated athlete-friendly terminology with active elite athletes. This co-production with elite athletes may have improved the accuracy of answering APSQ-J questions. However, we also recognize several limitations that may impact the findings. The current samples were all male professional rugby players and the response rate was less than 40%, although this is consistent with other mental health surveys in Japan. Future research should aim to examine the psychometric properties of the APSQ-J in a wider, more representative cohort, including female athletes and those from other sports. Furthermore, because we employed a cross-sectional survey design, the test–retest for the stability of the APSQ-J was not evaluated. Finally, the effectiveness of scale convergence and divergence could be better understood by consideration of critical stages of risk for athlete mental health, such as injury duration and severity and the transition from competitive sports.

## Conclusions

We have developed the APSQ-J, which enables screening for mental health status in Japanese elite athletes. Utilizing this scale may support athletes’ mental health via early detection of symptoms of psychological distress. There is increasing international interest in clinical practice and research related to mental health in the international sports society. APSQ-J, a brief and effective evaluation tool, is expected to be widely used in the Japanese sports society. In addition, early detection of psychological distress by this tool may help promote mental health support for elite athletes.

## Supplementary Information

**Additional file 1**. Comparison of Means and SDs of participants’ responses to APSQ between Japanese and Australian elite athletes.

## Data Availability

Data cannot be shared publicly because there exist ethical restrictions. Publishing data sets is not covered by the informed consent provided by the study participants, which was approved by the ethics committee of the Research Ethics Committee at the National Center of Neurology and Psychiatry. The data are not owned by a third party. Non-author contact information for the body imposing the restrictions upon the data, to which data access requests can be sent, is the Research Ethics Committee of the National Center of Neurology and Psychiatry (rinri-jimu@ncnp.go.jp).

## Abbreviations

APSQ: Athlete Psychological Strain Questionnaire
APSQ-J: Japanese version of the Athlete Psychological Strain Questionnaire
EFA: Exploratory factor analysis
CFA: Confirmatory factor analysis
K6: Kessler-6
SMHAT-1: Sports Mental Health Assessment Tool-1
IOC: International Olympic Committee
STROBE: Strengthening the Reporting of Observational Studies in Epidemiology
JRPA: Japan Rugby Players' Association
SD: Standard Deviation
RMSEA: Root mean square error of approximation
