# Supplementary material for: Validating a Japanese Version of the Athlete Psychological Strain Questionnaire
Source: Sports Med Open. 2021 Dec 11;7:90. doi: 10.1186/s40798-021-00385-9 (PMC8665958; doi:10.1186/s40798-021-00385-9)
Supplement: Supplementary file 1 — Additional file 1. Comparison of Means and SDs of participants’ responses to APSQ between Japanese and Australian elite athletes. [file 40798_2021_385_MOESM1_ESM.docx]

| Supplementary table 1.  Comparison of Means and SDs of participants’ responses to APSQ between Japanese and Australian elite athletes | | | | | |
| --- | --- | --- | --- | --- | --- |
|  |  | Current Japanese sample  (n = 219) | | Australian sample^22^ (n = 1007) | |
| Item no. | Statement | mean | sd | mean | sd |
| 1 | It was difficult to be around teammates | 1.50 | 0.77 | 1.35 | 0.69 |
| 2 | I found it difficult to do what I needed to do | 1.88 | 0.91 | 1.35 | 0.64 |
| 3 | I was less motivated | 2.16 | 1.03 | 1.59 | 0.85 |
| 4 | I was irritable, angry or aggressive | 1.74 | 0.88 | 1.50 | 0.77 |
| 5 | I could not stop worrying about injury or my performance | 2.65 | 1.25 | 1.65 | 0.93 |
| 6 | I found training more stressful | 1.94 | 1.02 | 1.61 | 0.87 |
| 7 | I found it hard to cope with selection pressures | 2.42 | 1.26 | 1.32 | 0.75 |
| 8 | I worried about life after sport | 2.47 | 1.24 | 1.93 | 1.1 |
| 9 | I needed alcohol or other substances to relax | 1.32 | 0.68 | 1.29 | 0.71 |
| 10 | It was difficult to be around teammates | 1.09 | 0.37 | 1.19 | 0.56 |
|  | Total score | 19.16 | 6.30 | 14.67 | 5.47 |
